# Supplementary material for: Fine-tuning RIPENING INHIBITOR (RIN) expression by introducing allelic mutations in its promoter using CRISPR/Cas9 multiplex editing
Source: Mol Hortic. 2026 Mar 2;6:17. doi: 10.1186/s43897-025-00200-z (PMC12952064; doi:10.1186/s43897-025-00200-z)
Supplement: Supplementary file 1 — Supplementary Material 1. [file 43897_2025_200_MOESM1_ESM.docx]

**SUPPLEMENTARY MATERIALS**

**Fine-tuning *RIPENING INHIBITOR* (*RIN)* expression by introducing allelic mutations in its promoter using CRISPR/Cas9 multiplex editing**

**Jiaqi Zhou^1,2^, Chiu-Ling Yang^1,3^, and Diane M. Beckles^1*^**

^1^Department of Plant Sciences, University of California, One Shields Avenue, Davis CA 95616, United States

^2^Cold Spring Harbor Laboratory, Cold Spring Harbor, NY, 11724, United States

^3^Agricultural Biotechnology Research Center, Academia Sinica, Nankang District, Taipei City, 115201, Taiwan

*Corresponding author. Email: dmbeckles@ucdavis.edu

Supplementary Table 1. Guide-RNA sequences used.

Supplementary Table 2. Primers used for gene construct assembly.

Supplementary Table 3. Primers used for screening and genotyping transformants.

Supplementary Table 4. RT-qPCR Primer set for *RIN* gene expression.

Supplementary Figure 1. RIN gene promoter DNA methylation status.

Supplementary Figure 2. Schematic of the CRISPR/Cas9 multiplex constructs.

Supplementary Figure 3. Summary of small indels position and sequence in the T_0_ (the first-generation) lines.

Supplementary Figure 4. Relative expression of *RIN* in red ripe fruit of WT and T_1_ (the second-generation) edited lines.

Supplementary Materials and Methods.

**Table S1. Guide-RNA sequences used**

| **Targeted sites** | **ID** | **Sequence (20bp, 5’-3’)** |
| --- | --- | --- |
| DMR-1 | gRNA-2 | CACCGCAACTTTCTTTTAAA |
| DMR-2 | gRNA-11 | CACCGCAACTTTCTTTTAAA |
| DMR-3 | gRNA-4 | AATTTTAACTACTAACTCGA |
| DMR-3 | gRNA-6 | AATTTGGGGAAGAAACGTCA |
| RIN bindings | gRNA-7 | GAGAACAAAGAACCATTAAA |
| RIN bindings | gRNA-8 | GCACTCTAAAAAAAGTTTAA |
| HY5 bindings | gRNA-9 | GTTTATGTATTATGCCCTCC |
| HY5 bindings | gRNA-10 | CATGTCATGCATGACACGTG |

**Table S2. Primers used for gene construct assembly**

| **Primer name** | **Sequence (5’-3’)** |
| --- | --- |
| CmYLCV | TGCTCTTCGCGCTGGCAGACATACTGTCCCAC |
| oCsy-E | TGCTCTTCTGACCTGCCTATACGGCAGTGAAC |
| oCsy4-B_gRNA2 | TCGTCTCCAAAGTTGCGGTGCTGCCTATACGGCAGTGAAC |
| orep-C_gRNA2 | TCGTCTCACTTTCTTTTAAAGTTTTAGAGCTAGAAATAGC |
| oCsy4-B_gRNA11 | TCGTCTCCGTGATATTGTCTCTGCCTATACGGCAGTGAAC |
| orep-C_gRNA3 | TCGTCTCATCACTAACAGGAGTTTTAGAGCTAGAAATAGC |
| oCsy4-B_gRNA4 | TCGTCTCCGTAGTTAAAATTCTGCCTATACGGCAGTGAAC |
| orep-C_gRNA4 | TCGTCTCACTACTAACTCGAGTTTTAGAGCTAGAAATAGC |
| oCsy4-B_gRNA6 | TCGTCTCCCTTCCCCAAATTCTGCCTATACGGCAGTGAAC |
| orep-C_gRNA6 | TCGTCTCAGAAGAAACGTCAGTTTTAGAGCTAGAAATAGC |
| oCsy4-B_gRNA7 | TCGTCTCAAGAACCATTAAAGTTTTAGAGCTAGAAATAGC |
| orep-C_gRNA7 | TCGTCTCCTTTTTAGAGTGCCTGCCTATACGGCAGTGAAC |
| oCsy4-B_gRNA8 | TCGTCTCCTTTTTAGAGTGCCTGCCTATACGGCAGTGAAC |
| orep-C_gRNA8 | TCGTCTCAAAAAAAGTTTAAGTTTTAGAGCTAGAAATAGC |
| oCsy4-B_gRNA9 | TCGTCTCCTAATACATAAACCTGCCTATACGGCAGTGAAC |
| orep-C_gRNA9 | TCGTCTCAATTATGCCCTCCGTTTTAGAGCTAGAAATAGC |
| oCsy4-B_gRNA10 | TCGTCTCCATGCATGACATGCTGCCTATACGGCAGTGAAC |
| orep-C_gRNA10 | TCGTCTCAGCATGACACGTGGTTTTAGAGCTAGAAATAGC |

**Table S3. Primers used for screening and genotyping transformants**

| **Primer name** | **Sequence (5’-3’)** |
| --- | --- |
| **Primers used in screening TDNA** | |
| Cas9_F | AGTACGTGACCGAGGGAATG |
| Cas9_R | GATCGTGGTAGGTTCCGAGA |
| TC320 | CTAGAAGTAGTCAAGGCGGC |
| M13F | GTAAAACGACGGCCAGT |
| **Primers used in screening mutations** | |
| RINp_+810F | TGCGATCACAGATACCCCTC |
| RINp_+161F | AGCTTGCCACGACTAGAGAA |
| RINp_5F | TGGTTCCCTAGTCAAGAAGAAAAGA |
| RINp_1106R | AAGTATCGGTCACTACTAAG |
| RINp_2F | GACCCTCAACTTTGGATGCG |
| RINp_2R | AAGTGCACCTTAAAGCTGGT |
| RINp_1947R | GCTCATCGAAAACTCCGATGC |
| RINp_1933R | CCGATGCACTAATGTTTGCCA |
| RINp_4R | GCTTTCAGCTTTCCAACGACA |

**Table S4. RT-qPCR Primer set for *RIN* gene expression (Ito et al., 2020)**

| **Primer name** | **Sequence (5’-3’)** |
| --- | --- |
| CAC_F | CCTCCGTTGTGATGTAACTGG |
| CAC_R | ATTGGTGGAAAGTAACATCATCG |
| RIN_F | GCTAGGTGAGGATTTGGGACAA |
| RIN_R | AATTTGCCTCAATGATGAATCCA |

**Figure S1. RIN gene promoter DNA methylation status**. The image of raw DNA methylation sites was from Lang *et al.* (Lang et al., 2017). The single-base level DNA methylation for fruit tissue was shown by the CG, CHG, and CHH contexts, respectively. The red panel (top) is for tomato fruit at the red ripe stage, the green panel (middle) is the data for fruit before the onset of ripening, and the blue (bottom) is for the Demethylase-*SlDML2* mutant. There are three DMRs identified and highlighted by the blue boxes. These regions are ripening-induced hypo-DMRs, because their DNA methylation levels drop during ripening. The sequence length of the DMRs is 218 bp, 45 bp and 201 bp, respectively.

**Figure S2. Schematic of the CRISPR/Cas9 multiplex constructs**. A gene construct containing (**A**) Two gRNAs for editing the RIN self-binding sites. (**B**) Two gRNAs for editing the HY5 binding sites. (**C**) Four gRNAs for editing the three hypo-DMRs that are described above. (**D**) Six gRNAs for the simultaneous targeting of the CREs to which RIN and HY5 bind, and the DMRs.

**Figure S3. Summary of the small indels (insertion or deletion) position and sequence in the T_0_ (the first-generation) lines**. Mutations were presented at (**A**) RIN binding site, (**B**) HY5 binding site, (**C**) DMRs region, (**D**) combination of RIN binding site, HY5 binding site, and DMR region. There are multiple alleles in some heterozygous or chimeric T_0_, represented by an addition of ‘ -1, -2, or -3’ after the T_0_ line’s identification number. The grey bars indicate the same sequence as WT, the white areas indicate ‘deletion’ regions, and the blue bars indicate those with ‘insertions.’ The pink bar at the top of each column indicates the gRNA targeting region (20 bp).

**Figure S4.** **Relative expression of *RIN* in red ripe fruit of WT and T_1_ (the second-generation) edited lines**. The x-axis indicates selected mutant genotypes and WT control. The y-axis indicates the log_2_ fold change relative to WT. Compared to WT, *P* < 0.01 is indicated by ‘**’, according to the one-way ANOVA test with Tukey’s multigroup correction.

**Materials and Methods**

1. **RIN gene promoter analysis**

The *RIN* promoter region was defined as the 2-3 kb region upstream of the *RIN* gene (NCBI ID: 543708) translation start site. The sequence was accessed through NCBI (JAAXDC010000005.1:5415385-5418385, *Solanum lycopersicum* cultivar Micro-Tom, chromosome 5, whole-genome shotgun sequence).

The sequence was used as input to identify potential cis-regulatory regions (CREs) in PlantCare (Lescot et al., 2002). The two identified CArG motifs to which RIN binds were identified according to (Bemer et al., 2012; Fujisawa et al., 2013). The DNA methylation status of the RIN gene promoter in ‘Micro-Tom’ was downloaded from (<https://www.ncbi.nlm.nih.gov/geo/query/acc.cgi?acc=GSE94903>) (Lang et al., 2017). The downloaded sequence data with DNA methylation in three contexts, i.e., CG, CHH and, CHG were imported to the Integrative genomics viewer (IGV) (Robinson et al., 2011). Comparing the DNA methylation levels in wild-type fruit at different ripening stages, the ripening-induced differential methylated regions (DMRs) were identified.

1. **Gene construct assembly**

The gRNAs targeting the three ripening-induced hypo-DMRs in the *RIN* promoter, two RIN self-binding sites (CArG motif), and HY5 binding sites were designed using CRISPOR (Concordet & Haeussler, 2018) and CRISPR-P (Lei et al., 2014). To avoid ‘off-targeting’, the gRNA sequences were selected based on the off-target mismatches score, followed by BLAST against the Micro-Tom genome through NCBI. The CRISPR/Cas9 constructs were generated using the pDIRECT_22C (Plasmid #91135, Addgene) backbone through Golden Gate cloning (Čermák et al., 2017). Some constructs carried gRNAs targeting a single type of site, while others carried multiple gRNAs to target different types of sites simultaneously. Assembled constructs were transformed into *E.coli* strain DH5-alpha. Plasmid DNA was isolated using a miniprep kit (Qiagen, Valencia, CA) and whole plasmid sequencing was done using the service offered by Plasmidsaurus (Eugene, OR, USA).

1. **Plant transformation and tissue culture**

Sequenced plasmids were transformed into *Agrobacterium tumefaciens* strain GV3101 using the heat-shock method (Goldbio, MO, USA). *A. tumefaciens-*mediated plant transformation was done as described in Albornoz *et al.* (Albornoz et al., 2023). Tomato tissue culture was performed under the guidance of the Plant Transformation Facility at UC Davis. The regenerated tomato plantlets with well-developed roots and shoots were transplanted into soil and hardened under controlled environmental conditions for two weeks. The adapted plants were transferred into the greenhouse at Davis, CA.

1. **Plant growth and fruit sampling**

All mutants and WT plants were grown in the greenhouse at UC Davis (Davis, CA, USA) using a completely random design (CRD). The UC ‘Mix C’ soil was placed in a 6.5-inch pot and watered (with liquid fertilizer) twice a day with a 3 min irrigation cycle. The greenhouse conditions were maintained at 25°C, with a 15-h light and 9-h dark cycle each day. The fruit were harvested and washed in 0.25% (v/v) sodium hypochlorite for three minutes, followed by rinsing with water and gently blotted until dry with paper towels. Fruit pericarps were sampled, frozen in liquid nitrogen and stored at -70°C for RNA isolation.

1. **Mutation detection**

The following genotyping standard procedures were applied for hundreds of regenerated first-generation (T_0_) plantlets. (1) Genomic DNA was extracted from young tomato leaves using a modified CTAB method (Zhou et al., 2021). (2) Genomic DNA quality was tested by a Nanodrop and a standard PCR amplification for a housekeeping gene *ACT7* using AmpliTaq (Applied biosystem, USA). (3) The genomic DNAs that passed the quality checks were used for screening T-DNA insertion by amplifying the Cas9 fragment of the construct. (4) Genomic DNA from individual lines with T-DNA insertions were used to amplify the gRNAs regions in the *RIN* promoter sequence, using flanking primers. (5) Direct Sanger sequencing was used to analyze the purified amplicons. The mutation type, i.e., homozygous, biallelic, heterozygous and chimeric (multiple mutations) was detected by NCBI blastn, TIDE (Brinkman et al., 2018) and ICE Synthego (Conant et al., 2022) together. Plants in the T_1_ generation were genotyped by PCR-amplifying the entire region targeted by the gRNAs, and subjecting the purified fragments to Sanger sequencing to detect small indels. To generate the aligned sequence figures, the sequence traces were first aligned in MEGA X software (Kumar et al., 2018), illustrated by the seqvisr and the ggmsa package (Charif & Lobry, 2007; Zhou et al., 2022) under the R environment.

1. **RNA isolation and RT-qPCR**

Fruit total RNA was isolated from around 100 mg fruit power using a Trizol-based protocol (Invitrogen, Thermofisher, USA). RNA quality and integrity were assessed by microvolume spectrophotometer and 0.8% (w/v) agarose gel electrophoresis. Around 500 ng total RNA was used as the input for the High-capacity Reverse Transcription kit (Thermofisher). The cDNA libraries were quality checked using a standard PCR (Amplitaq; Applied Biosystems). The RT-qPCR were performed according to Zhou *et al*. (Zhou et al., 2021), using the *SlCAC* gene as the internal control as reported (Ito et al., 2020).

1. **Fruit ripening speed assessment**

The five T_1_ generation lines assessed, included ‘A26A’, ‘A26B’, ‘A26H’, ‘A22H’, ‘A83’, and were compared to the WT. Ripening time was recorded as the duration between Breaker and Red Ripe fruit ripened on-the-vine. There were 114 WT fruit, 75 fruit for ‘A22H’, 57 fruit for ‘A26A’, 28 fruit for ‘A26H’, 24 fruit for ‘A26B’, and 32 fruit for ‘A83’, harvested from at least five tomato plants per genotype in this assay. All plants were grown simultaneously under the same conditions in the greenhouse at UC Davis in Summer 2023, and the data were recorded between 9-10 am daily.

1. **Fruit quality parameters assessment**

Fruit were harvested at Breaker, and stored at 20°C with 67% relative humidity under dark conditions for one week, to evaluate fruit quality. Fruit firmness was assessed using a Texture Analyzer (XT Plus, Texture 147 Technologies, Scarsdale, NY). The force was recorded when compressing the equatorial region of the whole fruit for 5 mm using a 2 mm flat stainless probe. The parameters were set as 2 mm/s for the pre-test speed, 1 mm/s for the test speed, followed by a 5 mm/s post speed. The average value was determined at three points for each fruit. Total Soluble Solids **(**TSS) was assayed by placing fruit juice in a refractometer at room temperature (Hanna Instruments, USA) (Zhou et al., 2021). The titratable acid (TA) was represented as grams of citric acid per 100 g of fresh weight.

1. **Statistical analysis**

A completely randomized design (CRD) was applied, in which treatment levels included genotype and ripening stages. Significant differences (*P* < 0.05) were determined by ANOVA or Student’s *t*-test in the R-environment.

**ReferenceS**

Albornoz, K., Zhou, J., & Beckles, D. M. (2023). Chemical induction of the Arabidopsis thaliana CBF1 gene in transgenic tomato fruit to study postharvest chilling injury. *Current Plant Biology*, *33*, 100275. <https://doi.org/https://doi.org/10.1016/j.cpb.2023.100275>

Bemer, M., Karlova, R., Ballester, A. R., Tikunov, Y. M., Bovy, A. G., Wolters-Arts, M., Rossetto, P. d. B., Angenent, G. C., & de Maagd, R. A. (2012). The Tomato FRUITFULL Homologs TDR4/FUL1 and MBP7/FUL2 Regulate Ethylene-Independent Aspects of Fruit Ripening. *The Plant Cell*, *24*(11), 4437-4451. <https://doi.org/10.1105/tpc.112.103283>

Brinkman, E. K., Kousholt, A. N., Harmsen, T., Leemans, C., Chen, T., Jonkers, J., & van Steensel, B. (2018). Easy quantification of template-directed CRISPR/Cas9 editing. *Nucleic Acids Research*, *46*(10), e58-e58. <https://doi.org/10.1093/nar/gky164>

Čermák, T., Curtin, S. J., Gil-Humanes, J., Čegan, R., Kono, T. J. Y., Konečná, E., Belanto, J. J., Starker, C. G., Mathre, J. W., Greenstein, R. L., & Voytas, D. F. (2017). A Multipurpose Toolkit to Enable Advanced Genome Engineering in Plants. *The Plant Cell*, *29*(6), 1196-1217. <https://doi.org/10.1105/tpc.16.00922>

Charif, D., & Lobry, J. R. (2007). SeqinR 1.0-2: A Contributed Package to the R Project for Statistical Computing Devoted to Biological Sequences Retrieval and Analysis. In U. Bastolla, M. Porto, H. E. Roman, & M. Vendruscolo (Eds.), *Structural Approaches to Sequence Evolution: Molecules, Networks, Populations* (pp. 207-232). Springer Berlin Heidelberg. <https://doi.org/10.1007/978-3-540-35306-5_10>

Conant, D., Hsiau, T., Rossi, N., Oki, J., Maures, T., Waite, K., Yang, J., Joshi, S., Kelso, R., Holden, K., Enzmann, B. L., & Stoner, R. (2022). Inference of CRISPR Edits from Sanger Trace Data. *The CRISPR Journal*, *5*(1), 123-130. <https://doi.org/10.1089/crispr.2021.0113>

Concordet, J.-P., & Haeussler, M. (2018). CRISPOR: intuitive guide selection for CRISPR/Cas9 genome editing experiments and screens. *Nucleic Acids Research*, *46*(W1), W242-W245. <https://doi.org/10.1093/nar/gky354>

Fujisawa, M., Nakano, T., Shima, Y., & Ito, Y. (2013). A Large-Scale Identification of Direct Targets of the Tomato MADS Box Transcription Factor RIPENING INHIBITOR Reveals the Regulation of Fruit Ripening. *The Plant Cell*, *25*(2), 371-386. <https://doi.org/10.1105/tpc.112.108118>

Ito, Y., Sekiyama, Y., Nakayama, H., Nishizawa-Yokoi, A., Endo, M., Shima, Y., Nakamura, N., Kotake-Nara, E., Kawasaki, S., Hirose, S., & Toki, S. (2020). Allelic Mutations in the Ripening -Inhibitor Locus Generate Extensive Variation in Tomato Ripening1. *Plant Physiology*, *183*(1), 80-95. <https://doi.org/10.1104/pp.20.00020>

Kumar, S., Stecher, G., Li, M., Knyaz, C., & Tamura, K. (2018). MEGA X: Molecular Evolutionary Genetics Analysis across Computing Platforms. *Molecular Biology and Evolution*, *35*(6), 1547-1549. <https://doi.org/10.1093/molbev/msy096>

Lang, Z., Wang, Y., Tang, K., Tang, D., Datsenka, T., Cheng, J., Zhang, Y., Handa, A. K., & Zhu, J.-K. (2017). Critical roles of DNA demethylation in the activation of ripening-induced genes and inhibition of ripening-repressed genes in tomato fruit. *Proceedings of the National Academy of Sciences*, *114*(22), E4511-E4519. <https://doi.org/10.1073/pnas.1705233114>

Lei, Y., Lu, L., Liu, H.-Y., Li, S., Xing, F., & Chen, L.-L. (2014). CRISPR-P: A Web Tool for Synthetic Single-Guide RNA Design of CRISPR-System in Plants. *Molecular Plant*, *7*(9), 1494-1496. <https://doi.org/10.1093/mp/ssu044>

Lescot, M., Déhais, P., Thijs, G., Marchal, K., Moreau, Y., Van de Peer, Y., Rouzé, P., & Rombauts, S. (2002). PlantCARE, a database of plant cis-acting regulatory elements and a portal to tools for in silico analysis of promoter sequences. *Nucleic Acids Research*, *30*(1), 325-327. <https://doi.org/10.1093/nar/30.1.325>

Robinson, J. T., Thorvaldsdóttir, H., Winckler, W., Guttman, M., Lander, E. S., Getz, G., & Mesirov, J. P. (2011). Integrative genomics viewer. *Nature Biotechnology*, *29*(1), 24-26. <https://doi.org/10.1038/nbt.1754>

Zhou, J., Chen, B., Albornoz, K., & Beckles, D. M. (2021). Postharvest handling induces changes in fruit DNA methylation status and is associated with alterations in fruit quality in tomato (Solanum lycopersicum L.). *Scientia Horticulturae*, *283*, 110090. <https://doi.org/https://doi.org/10.1016/j.scienta.2021.110090>

Zhou, L., Feng, T., Xu, S., Gao, F., Lam, T. T., Wang, Q., Wu, T., Huang, H., Zhan, L., Li, L., Guan, Y., Dai, Z., & Yu, G. (2022). ggmsa: a visual exploration tool for multiple sequence alignment and associated data. *Briefings in Bioinformatics*, *23*(4), bbac222. <https://doi.org/10.1093/bib/bbac222>
